# Relationship between lung function impairment, hypertension, and major adverse cardiovascular events: A 10‐year follow‐up study

**DOI:** 10.1111/jch.14364

**Published:** 2021-09-16

**Authors:** Jiaqi Wang, He Dai, Chong Chen, Ganling Ding, Yongqing Zhang, Yu Qin, Yuqing Zhang, Quanyong Xiang

**Affiliations:** ^1^ School of Public Health Southeast University Nanjing Jiangsu Province China; ^2^ Department of Cardiology Nanjing Jiangning Hospital The Affiliated Jiangning Hospital of Nanjing Medical University Nanjing Jiangsu Province China; ^3^ Department of Chronic Non‐communicable Disease Control Jiangsu Province Center for Disease Control and Prevention Nanjing Jiangsu Province China

**Keywords:** cardiovascular disease, hypertension, lung function impairment, stroke

## Abstract

Lung function impairment and hypertension, especially hypertension, are risk factors of major adverse cardiovascular events (MACEs). However, the relationships among lung function impairment, hypertension, and MACEs have not been well‐reported. We aimed to investigate the association between lung function and hypertension and MACEs. We studied 6769 people who were a representative sample of the general population in Jiangsu Province using the multi‐stage stratified cluster sampling method. The average age was 51.54 years. Cox proportional hazards models were used to analyze the relationships between the blood pressure status and various types of lung function impairment related to MACEs. Over a follow‐up of 10 years, 236 MACEs occurred. After adjusting for age, sex, BMI, smoking, drinking, education, physical activity, diabetes mellitus, dyslipidemia, creatine and use of antihypertensive drugs, hypertension [hazard ratio (HR) = 2.154, 95% confidence intervals (CI): 1.565–2.966], and restrictive lung function impairment (RLFI) (HR = 1.398, 95% CI: 1.021–1.879) were independently associated with MACEs. Individuals with hypertension and RFLI had the highest risk for MACEs (HR = 2.930, 95% CI: 1.734–4.953) and stroke (HR = 3.296, 95% CI: 1.862–5.832). Moreover, when combined with hypertension, obstructive lung function impairment (OLFI) (HR = 2.376, 95% CI: 1.391–4.056) and mixed lung function impairment (MLFI) (HR = 2.423, 95% CI: 1.203–4.882) were associated with MACEs. There is a synergistic effect of lung function impairment (especially RLFI) and hypertension on MACEs. Therefore, more attention should be paid to the incidence of MACEs in individuals with impaired lung function, especially those who have hypertension.

## 1 BACKGROUND

Cardiovascular disease comprises heart and vascular disease and is the main cause of global mortality and disability. In developing countries, the social and economic burden due to major adverse cardiovascular events (MACEs) is increasing yearly.^1^ In 2016, cardiovascular mortality was the main cause of death, accounting for more than 40% of deaths in the Chinese population, and its rate was higher than that of tumors and other diseases.^2^ According to the China Cardiovascular Health and Disease Report in 2019, 330 million people suffer from MACEs, among which stroke ranked first with 13 million people.^3^ Hypertension is a risk factor for MACEs. A previous study showed that when blood pressure exceeded 115/75 mmHg, the risk of MACEs would double with each increase of 20/10 mmHg.^4^ Previous studies have shown that the risk of stroke among hypertensive patients was seven times more than those with normal blood pressure.^5^ In addition, some cross‐sectional studies have shown that reduced lung function is associated with an increased risk of MACEs.^6^, ^7^ In particular, several studies have shown a significant negative association between lung function and ischemic stroke.^8^, ^9^ Previous research has used continuous variables of forced vital capacity (FVC), forced expiratory volume in 1 s (FEV_1_), and percent predicted FEV_1_ to evaluate the relationship between lung function and MACEs. Few follow‐up studies in cohorts have examined the association between different types of lung function impairment and MACEs,^10^, ^11^ especially the combination of hypertension and different types of lung function impairment related to MACEs.

Therefore, this study aimed to examine the association between different types of lung function impairment combined with hypertension and MACEs. Lung function, blood pressure, and other information were collected at baseline. Lung function impairment was classified as restrictive lung function impairment (RLFI), obstructive lung function impairment (OLFI), and mixed lung function impairment (MLFI). The participants were followed up for approximately 10 years during which cardiovascular events were investigated.

## 2 METHODS

### 2.1 Population

A multi‐stage, stratified sampling method (community, household, individual) was used to select a representative sample of general participants from Jiangsu Province, China. The first stage was based on geographical regions (North, Midland, and South Jiangsu Province), the second stage was based on urban/rural locations, and the third stage was based on the socioeconomic status. Finally, six counties and six districts were selected in this study. Four sub‐districts or four towns from each district or county were further randomly selected. Two neighborhood communities or two administrative villages from each sub‐district or town were then selected according to the random number table. Eighty households were randomly chosen from each selected neighborhood community or administrative village. Finally, we randomly selected one eligible person, according to the standard protocol, who was aged ≥35 and ≤70 years and had been living in their current residence for at least 6 months from each selected household using the Kish selection table. Finally, 7680 participants were recruited. Demographic information, and physical and laboratory examination results were collected from baseline in October 2007 to October 2008. Eligible participants for selection met the following criteria: (1) individuals who were aged between 35 and 70 years and (2) individuals who intended to remain in the current address for a further 4 years. Exclusion criteria in this study were as follows: (1) individuals with a history of MACEs; (2) individuals who were physically unfit for lung function tests or unwilling to cooperate with lung function tests; and (3) individuals who were physically unfit for blood sampling or unwilling to cooperate with blood sampling.

A total of 131, 138, 169, and 185 participants were excluded because of missing baseline information, insufficient laboratory data, a history of MACEs, and failure to complete lung function tests, respectively. During follow‐up, 288 individuals did not have events and timing recorded, and they were also excluded from statistical analysis owing to a lack of follow‐up information. A total of 6769 eligible individuals aged 35–70 years were enrolled as the final study sample (3858 men and 2911 women).

Written informed consent was obtained from all participants. The study protocol was approved by the ethical review committee of the Jiangsu Province Center for Disease Control and Prevention. Data of individuals are not shown in any form (including any individual details, images, or videos) in this manuscript. The procedures were in accordance with the standards of the ethics committee of Jiangsu Provincial Center for Disease Control and Prevention and with the Declaration of Helsinki (1975, revised 2013).

### 2.2 Data collection

A standardized questionnaire was used to collect demographic information, education level, physical activity level, smoking and drinking habits, presence of diabetes and hypertension and other factors. The Omron digital blood pressure measuring device [Omron HEM‐757, Omron Medical Equipment (Beijing) Co., Ltd] was used for blood pressure measurement. A 12‐h fasting blood sample was collected to measure levels of fasting blood glucose (FBG), serum total cholesterol (TC), serum creatinine, serum triglycerides (TG), serum low density lipoprotein cholesterol (LDL‐C), and serum high‐density lipoprotein cholesterol (HDL‐C). A 10 mL fasting blood sample is collected from all consenting participants. Blood samples were centrifuged and transferred to a centralized long‐term storage in secure −70°C freezers or large −180°C liquid nitrogen tanks for later testing. FBG levels were measured by an enzymatic method. TC, TG, HDL‐C, and LDL‐C levels were measured with the Boehringer‐Mannheim Diagnostics High‐Performance enzymatic reagent (Boehringer‐Mannheim, Indianapolis, IN, USA) and the ABA 200 bichromatic analyzer (Abbott Laboratories, Chicago, IL, USA). FVC and FEV_1_ were measured with a portable spirometer (MicroGP; MicroMedical, Chatham, IL, USA) after inhalation of post‐bronchodilators, without spirographs using a standardized protocol. We conducted follow‐up every 3 years after baseline for outcomes of MACEs, which comprised stroke, myocardial infarction, and heart failure, and the follow‐up was completed by December 2018.

### 2.3 Definitions

Smoking was defined as a history of smoking and smoking at least one cigarette per day in the past year.^12^ Drinking was defined as having alcohol more than once per month in the past year.^13^ Physical activity levels were assessed with the International Physical Activity Questionnaire.^14^, ^15^ According to the types and frequency of physical activity in 1 week, we calculated the metabolic equivalent task (MET), and divided physical activity into three levels of low physical activity (< 600 MET min/week), moderate physical activity (physical activity ≥600 and < 3000 MET min/week) and high physical activity (≥3000 MET min/week). Hypertension was defined by self‐reported or a measured blood pressure > 140/90 mmHg or taking antihypertensive drugs. Dyslipidemia was defined as TC ≥6.22 mmol/L, or HDL‐C < 1.04 mmol/L, or LDL‐C ≥4.14 mmol/L, or TG ≥2.26 mmol/L.^16^ Normal lung function was defined as an FVC ≥80% of the predicted FVC and FEV_1_/FVC ≥0.7. RLFI was defined as an FVC < 80% of the predicted FVC and FEV_1_/FVC ≥0.7. OLFI was defined as an FVC ≥80% of the predicted FVC and FEV_1_/FVC < 0.7. MLFI was defined as an FVC < 80% of the predicted FVC and FEV_1_/FVC < 0.7. We used the following formula to calculate the predicted FVC: predicted FVC = 0.04669 (height)+0.45229 (sex)−0.01326 (age)+0.01664 (weight)−4.79287.^17^ Outcome events for myocardial infarction, stroke and heart failure were determined by hospital diagnosis under certain criteria.^18^

### 2.4 Statistical analysis

The population was divided into eight groups according to their blood pressure and lung function status, and baseline characteristics in each group in relation to MACEs were analyzed. The mean with standard deviation was used to represent quantitative data. Analysis of variance was used to compare differences between groups using individuals with hypertension and normal lung function as the reference group. The frequency and the percentage were used to represent qualitative data. The χ^2^ test was used to compare differences between groups using individuals with no hypertension and normal lung function as the reference group. The crude incidence rate (per 1000 person‐years) of MACEs (stroke, myocardial infarction, and heart failure) during follow‐up were calculated. When calculating the incidence, we recorded two or more MACEs (stroke, myocardial infarction, or heart failure) events in the same person as two or more MACEs. The Cox proportional hazard model was used to analyze the effect of hypertension only, lung function impairment status only in two models (Model 1 was adjusted for age and sex. Model 2 was adjusted for age, sex, BMI, smoking, drinking, education, physical activity, diabetes mellitus, dyslipidemia, creatine, and use of antihypertensive drugs), and the combination of these two factors on MACEs and stroke after adjusting for age, sex, BMI, smoking, drinking, education, physical activity, diabetes mellitus, dyslipidemia, creatine, and use of antihypertensive drugs. The single factor of stroke was selected because it has the highest morbidity and mortality rates among MACEs in China, and the number of stroke cases in our study also accounted for the largest proportion of MACEs. In the Cox model, we counted the participants’ first MACE as the outcome of concern. Hazard ratios (HRs) and 95% confidence intervals (CIs) were used to indicate the hazard of MACE in different lung impairment and hypertension status. In order to eliminate the effect of antihypertensive drugs on lung function, COX regression was carried out again after excluding the subjects taking antihypertensive drugs.

## 3 RESULTS

### 3.1 Characteristics of the study participants

Table 1 shows the baseline characteristics of the study participants. The mean age was 51.54±9.31 years (range: 35–70 years) and 2911 (43.00%) were men. A total of 2613 (38.60%) participants were diagnosed with hypertension, 2791 (41.23%) were classified as normal lung function, 1697 (25.07%) were classified as RLFI, 1920 (28.36%) were classified as OLFI, and 361 (5.33%) were classified as MLFI. There were 1857 (27.43%) participants who were free of hypertension and lung function impairment. There were 164 (2.42%) participants who had MLFI and were free of hypertension. Compared with participants who had normal lung function and no hypertension as the reference group, those in the other groups were older (*p *< .001), had a higher body mass index (BMI) (*p *< .001), and had a higher rate of diabetes (*p *< .05). The numbers of smokers and drinkers with hypertension and MLFI were significantly higher than those in the reference group (both *p *< .05). The education level, the physical activity level, and laboratory serological indicators in the other groups were different compared with the reference group.

**TABLE 1:** Characteristics of 6769 participants according to hypertension and lung function impairment status

	Non‐hypertension				Hypertension			
Characteristics	normal lung function (no. = 1857)	RLFI (no. = 909)	OLFI (no. = 1226)	MLFI (no. = 164)	normal lung function (no. = 934)	RLFI (no. = 788)	OLFI (no. = 694)	MLFI (no. = 197)
Age, yr	47.80±8.75	50.08±8.74^b^	50.01±9.11^b^	52.43±9.16^b^	54.06±8.88^b^	55.94±8.46^b^	55.48±8.17^b^	58.88±9.31 ^b^
Male sex, no. (%)	994(53.53)	356(39.16)^b^	236(19.25)^b^	121(73.78)^b^	543(58.14)^a^	335(42.51)^b^	186(26.80)^b^	140(70.07)^b^
BMI, kg/m^2^	23.25±3.04	25.27±5.09^b^	23.29±3.39	24.46±7.41^b^	24.73±3.10^b^	27.01±5.36^b^	24.73±3.78^b^	26.62±6.04^b^
Smoking, no. (%)	699(37.64)	222(24.42)^b^	164(13.38)^b^	72(43.90)	381(40.79)	215(27.28)^b^	130(18.73)^b^	99(50.25)^a^
Drinking, no. (%)	514(27.68)	163(17.93)^b^	112(9.13)^b^	36(21.95)	336(35.97)^b^	188(23.86)^a^	111(15.99)^b^	73(37.06)^a^
Education, no. (%)								
None, Primary, or Unknown	477(25.69)	312(34.32)^b^	416(33.93)^b^	43(26.22)	341(36.51)^b^	333(42.26)^b^	321(46.26)^b^	89(45.18)^b^
Secondary/High/Higher secondary	1321(71.13)	561(61.72)	784(63.95)	112(68.29)	567(60.71)	425(53.93)	348(50.14)	95(48.22)
Trade or College/University	59(3.18)	36(3.96)	26(2.12)	9(5.49)	26(2.78)	30(3.81)	25(3.60)	13(6.60)
Physical activity, no. (%)								
Low	386(20.79)	168(18.48)	186(15.17)^b^	27(16.46)	197(21.09)	174(22.08)^a^	129(18.59)	35(17.77)
Moderate	812(43.72)	438(48.19)	598(48.78)	68(41.46)	405(43.36)	390(49.49)	334(48.13)	99(50.25)
High	659(35.49)	303(33.33)	442(36.05)	69(42.07)	332(35.55)	224(28.43)	231(33.29)	63(31.98)
Diabetes Mellitus, no. (%)	31(1.67)	30(3.30)^a^	24(1.96)	6(3.66)	32(3.43)^a^	62(7.87)^b^	40(5.76)^b^	10(5.08)^a^
Glucose, mmol/L	5.43±1.09	5.70±1.31^b^	5.43±1.13	5.38±1.24	5.82±1.55^b^	6.18±1.92^b^	5.90±1.59^b^	6.20±1.82^b^
Dyslipidemia, no. (%)	522(28.11)	224(24.64)	336(27.41)	56(34.15)	328(35.12)^b^	267(33.88)^a^	214(30.84)	68(34.52)
Cholesterol, mmol/L	4.37±0.83	4.47±0.91^a^	4.38±0.89	4.35±0.96	4.54±0.84^b^	4.75±0.96^b^	4.58±0.88^b^	4.48±0.89
Triglyceride, mmol/L	1.33±0.94	1.38±1.09	1.35±1.05	1.51±1.20	1.64±1.20^b^	1.81±1.53^b^	1.71±1.34^b^	1.84±1.30^b^
HDL‐C, mmol/L	1.31±0.31	1.34±0.30^a^	1.32±0.33	1.23±0.28^a^	1.32±0.34	1.35±0.32^a^	1.35±0.33^a^	1.29±0.30^a^
LDL‐C, mmol/L	2.45±0.65	2.50±0.68^b^	2.41±0.73	2.40±0.77	2.47±0.70	2.59±0.75^b^	2.44±0.74	2.29±0.80^a^
Using antihypertensive drugs, no. (%)	–	–	–	–	283(30.30)	319(40.48)^d^	228(32.85)	85(43.15)^d^
ACEI/ARB, no. (%)	–	–	–	–	43(4.93)	38(4.82)	21(3.03)	14(7.11)
Beta blocker, no. (%)	–	–	–	–	9(0.96)	14(1.78)	4(0.58)	4(2.03)
Calcium antagonist, no. (%)	–	–	–	–	58(6.21)	57(7.23)	56(8.07)	16(8.12)
Diuretic, no. (%)	–		–	–	140(14.99)	167(21.19)^c^	127(18.30)	50(25.38)^c^
Other, no. (%)	–	–	–	–	33(3.53)	43(5.46)	20(2.88)	1(0.51)
Creatine, μmol/L	91.21±15.07	88.95±17.79^a^	82.29±15.80^b^	86.41±21.19^a^	93.60±15.46^a^	90.22±17.80	85.82±19.50^b^	96.28±34.12^b^

*Note*: data are expressed as number (%) or mean ± standard deviation. Individuals without hypertension and with normal lung function were used as the reference group. ^a^ *p *< .05; ^b^ *p *< .001 compared with the reference group; ^c^ *p *< .05; ^d^ *p *< .001 compared with the group of individuals with hypertension and normal lung function.

### 3.2 Incidence rate of MACEs according to hypertension and the lung function status during follow‐up

During the follow‐up of 10 years, 232 individuals had 236 incident MACEs (4.24/1000 person‐years), which comprised 190 strokes (3.41/1000 person‐years), 41 myocardial infarctions (0.73/1000 person‐years), and five heart failures (0.09/1000 person‐years). Table 2 shows the crude incidence rate of MACEs and stroke according to the presence of hypertension and the lung function impairment status. Participants with hypertension and MLFI had the highest incidence rate of MACEs (9.26/1000 person‐years), followed by participants with hypertension and RLFI (8.96/1000 person‐years). Participants with hypertension and RLFI had the highest incidence rate of stroke (8.02/1000 person‐years), followed by participants with hypertension and OLFI (5.78/1000 person‐years).

**TABLE 2:** Incidence rate of MACEs according to hypertension and lung function impairment status

		No. of events/Total No.	Person‐years	Crude Rate
Incident of MACEs (No. = 236)				
Non‐hypertension	normal lung function	23/1857	15262	1.51
	RLFI	23/909	7471	3.08
	OLFI	16/1226	10076	1.59
	MLFI	3/164	1348	2.23
Hypertension	normal lung function	57/934	7676	7.43
	RLFI	58/788	6476	8.96
	OLFI	41/694	5704	7.19
	MLFI	15/197	1619	9.26
Incident of stroke (No. = 190)				
Non‐hypertension	normal lung function	19/1857	15283	1.24
	RLFI	21/909	7481	2.81
	OLFI	13/1226	10090	1.29
	MLFI	1/164	1350	0.74
Hypertension	normal lung function	42/934	7687	5.46
	RLFI	52/788	6485	8.02
	OLFI	33/694	5712	5.78
	MLFI	9/197	1621	5.55

### 3.3 HRs of MACEs and stroke according to hypertension or lung function impairment status

Table 3 shows the HRs of MACEs and stroke according to hypertension or the lung function impairment status. The HRs of MACEs and stroke were 2.154 (95% CI: 1.565–2.966) and 2.125 (95% CI: 1.494–3.021), respectively, in participants with hypertension compared with those without hypertension when all cardiovascular risk factors were adjusted for. Participants with RLFI had higher HRs of MACEs (HR = 1.398, 95% CI: 1.021–1.879) and stroke (HR = 1.606, 95% CI: 1.126–2.289) compared with those with normal lung function as the reference when all cardiovascular risk factors were adjusted for in Model 2. Participants with MLFI also had a higher HR (HR = 2.102, 95% CI: 1.244–3.552) compared with the reference group when unadjusted for other factors. Differences in HRs of stroke between OLFI, MLFI, and the reference group were not significant.

**TABLE 3:** HRs of MACES according to hypertension or lung function impairment status

	Hypertension		Lung Function			
Variable	no	yes	normal lung function	RLFI	OLFI	MLFI
Incident MACEs						
Unadjusted	1.000	4.068(3.054‐5.419)	1.000	1.941(1.420‐2.654)	1.050(0.746‐1.478)	2.102(1.244‐3.552)
Model 1	1.000	2.460(1.828‐3.311)	1.000	1.495(1.090‐2.051)	0.887(0.627‐1.256)	1.194(0.702‐2.029)
Model 2	1.000	2.154(1.565‐2.966)	1.000	1.398(1.021‐1.879)	0.911(0.643‐1.291)	1.034(0.603‐1.773)
Incident stroke						
Unadjusted	1.000	3.978(2.902‐5.453)	1.000	2.288(1.627‐3.219)	1.082(0.738‐1.587)	1.579(0.809‐3.083)
Model 1	1.000	2.421(1.746‐3.358)	1.000	1.760(1.247‐2.484)	0.913(0.619‐1.348)	0.908(0.619‐1.348)
Model 2	1.000	2.125(1.494‐3.021)	1.000	1.606(1.126‐2.289)	0.928(0.628‐1.370)	0.805(0.407‐1.591)

*Note*: Model 1 was adjusted for age and sex. Model 2 was adjusted for age, sex, BMI, smoking, drinking, education, physical activity, diabetes mellitus, dyslipidemia, creatine, and use of antihypertensive drugs. *p* values for testing differences in HRs of MACEs or stroke were analyzed used the categories of participants without hypertension and those with normal lung function as the reference groups.

### 3.4 HRs of MACEs and stroke according to the presence of hypertension and lung function impairment status

**FIGURE 1:**
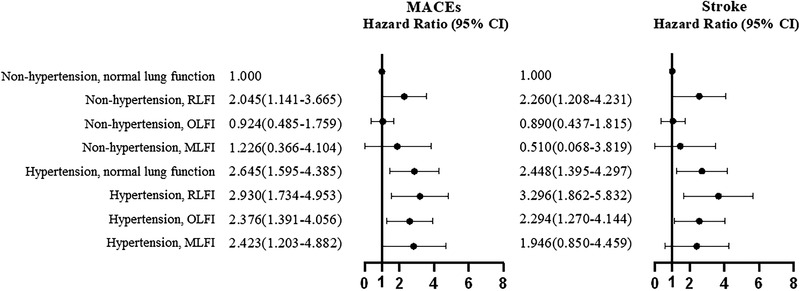
HRs of MACEs according to the presence of hypertension and lung function impairment status. Hazard ratios are presented after adjusting for age, sex, BMI, smoking, drinking, education, physical activity, diabetes mellitus, dyslipidemia, creatine and use of antihypertensive drugs. Individuals without hypertension and normal lung function were the reference group

The lung function impairment status and hypertension were combined in HR analysis (Figure 1). Participants with hypertension and RLFI had the highest HR for MACEs (HR = 2.930, 95% CI: 1.734–4.953) and stroke (HR = 3.296, 95% CI: 1.862–5.832). When combined with hypertension, OLIF (HR = 2.376, 95% CI: 1.391–4.056) and MLFI (HR = 2.423, 95% CI: 1.203–4.882) also increased the HRs of MACEs. After excluding subjects taking antihypertensive drugs, the results were similar, as shown in Table 4.

**TABLE 4:** HRs of MACEs according to the presence of hypertension and lung function impairment status excluding individuals using antihypertensive drugs (sensitivity analysis) (No. = 5854)

	MACEs	Stroke
Non‐hypertension, normal lung function	1.000	1.000
Non‐hypertension, RLFI	1.806(1.041‐3.358)	1.837(1.148‐3.161)
Non‐hypertension, OLFI	0.946(0.459‐1.950)	0.981(0.510‐1.887)
Non‐hypertension, MLFI	0.546(0.072‐4.147)	1.332(0.394‐4.508)
Hypertension, normal lung function	2.675(1.408‐5.085)	2.567(1.423‐4.633)
Hypertension, RLFI	2.769(1.533‐5.002)	2.944(1.729‐5.015)
Hypertension, OLFI	1.688(1.209‐3.125)	1.867(1.197‐3.183)
Hypertension, MLFI	2.170(1.199‐4.960)	1.662(0.654‐4.223)

*Note*: hazard ratios are presented after adjusting for age, sex, BMI, smoking, drinking, education, physical activity, diabetes mellitus, dyslipidemia, and creatine. Individuals without hypertension and normal lung function were the reference group.

## 4 DISCUSSION

In this prospective study, the main findings were an association between hypertension or RLFI and MACEs or stroke. After adjusting for potential confounders, individuals who had high blood pressure and RLFI had the highest risk for MACEs. However, there was no significant association between only OLFI or only MLFI and MACEs.

In 2016, there were 4.34 million deaths from MACEs in China, including 2.10 million deaths from stroke, which was the main cause of death.^15^, ^19^ The prevalence of MACEs continues to rise, with more than 290 million cases of MACEs in China.^2^ The high prevalence and mortality rates of MACEs impose a great economic and social burden. Therefore, there is an urgent need to examine cardiovascular risk factors and to control and prevent them.

Our study showed that hypertension was associated with an increased risk of major MACEs and stroke. Hypertension is a recognized risk factor for MACEs and is independent of other risk factors. The risks of myocardial infarction, stroke and heart failure increase with an increase in blood pressure.^4^ A review of 15 clinical trials showed that antihypertensive therapy reduced the risk of stroke, myocardial infarction and heart failure by 35% to 40%, 20% to 25%, and > 50%, respectively.^20^

The current study also showed that lung function impairment, especially RLFI, increased the risk of MACEs. In the definition of FVC < 80% of the predicted FVC and FEV_1_/FVC ≥0.7, RLFI mainly manifests as a reduction in FVC, with no obvious change in FEV_1_. FVC refers to the maximum volume of air that can be exhaled as soon as possible after maximum inhalation. In normal conditions, FVC is slightly lower than that measured without time constraints. FEV_1_ refers to the amount of air that can be exhaled in 1 s as soon as possible after maximum inhalation and it is usually expressed as the percentage of FEV in FVC at the end of the first second. Clinically, FEV_1_/FVC is also the primary marker to distinguish between OLFI and RLFI. In patients with RLFI, such as pulmonary fibrosis, FEV_1_ and FVC are decreased, but FEV_1_/FVC remains at a normal value.^21^ A previous study showed that decreased FVC was prevalent in individuals with MACEs.^22^ A study by Honda and associates suggested that individuals with RLFI had a high HR for cardiovascular death (HR = 2.61, 95% CI: 1.22–5.21) during a 10‐year follow‐up, but in individuals with OLFI or MLFI, this association was not found.^23^ Another study used the Framingham risk score to evaluate the relationship between FVC and mortality.^24^ This previous study showed that individuals with a low FVC had an almost three‐fold HR for all‐cause mortality than those with a normal FVC in the intermediate Framingham risk score group. In another study on a large sample of Caucasians, OLFI, but not RLFI, was associated with hospitalization for MACEs, although both were associated with cardiovascular death.^25^ These findings are different to those in our study. A prospective study showed that RLFI and OLFI were independent risk factors for incident MACEs.^26^ There are large individual differences in vital capacity, which is related to body size, sex, age, body position, and respiratory muscle strength.^21^ Therefore, the differences in the results of different studies may have been caused by different population characteristics. Several previous studies have suggested that metabolic syndrome is associated with RLFI,^27^, ^28^ which may explain part of the association between lung function and MACEs. Additionally, other studies have shown that RLFI is associated with an increased risk of atherosclerosis,^29^ which is a recognized underlying cause of MACEs, and the specific mechanism may be related to systemic inflammation.^30^

This study showed an increased risk of MACEs in individuals with both hypertension and lung function impairment, regardless of the type of lung function impairment, which has rarely been reported.^10^, ^11^ This study also showed that the HRs of MACEs and stroke in individuals with only hypertension were 2.344 and 2.300, respectively, which were higher than the HR in individuals with only RLFI. Additionally, although OLFI or MLFI was not significantly associated with the occurrence of MACEs or stroke, the HR of these two types of lung function impairment with hypertension was higher than that in individuals with only hypertension. This finding suggested an association between blood pressure and lung function. Previous studies have shown that low lung function was associated with a high future blood pressure and the occurrence of MACEs.^31^, ^32^ The prospective study results of Sparrow^33^ and Tockman^34^ showed that baseline FVC was significantly negatively correlated with the risk of hypertension, and was independent of alcohol consumption, smoking, salt addiction, and other factors. FVC is an important predictor of hypertension and may affect the morbidity and mortality of MACEs through hypertension. Therefore, hypertension may be an important link between lung function and MACEs. On the other hand, hypertension affects the structure and function of the lungs and pulmonary circulation. The important pathological feature of hypertension is the increase of peripheral vascular resistance, which leads to the decrease of blood supply, small airway injury, muscle endurance in the region under its jurisdiction, and the decrease of lung function through the influence of small airway ventilation.^35^ Hypertension and lung function impairment interact with each other, and their association with MACEs may be based on systemic inflammation and endothelial dysfunction.^36^

Our study has some strengths, which were its prospective nature with a large sample, and the follow‐up was long. Additionally, we classified lung function impairment and combined it with hypertension to investigate the outcome of MACEs in different types of lung function impairment. However, this study also has some limitations. We did not identify the sequence of hypertension and lung function impairment, and lack evidence to establish a clear association between hypertension and lung function impairment. Therefore, the role of lung function impairment and hypertension in the occurrence of MACEs, as well as the mechanism of changes in lung function affecting the occurrence of cardiovascular events, require further research.

In conclusion, in this cohort study, hypertension and RLFI were associated with MACEs, especially stroke. This finding provides a new reference for prevention and control of MACEs. In addition to measuring and monitoring blood pressure, indicators, such as FVC and FEV_1_, should be monitored. For individuals with impaired lung function, especially those with hypertension, attention should be paid to relevant control to reduce the incidence of MACEs in the future.

## CONFLICT OF INTEREST

The authors declare no conflict of interest.

## AUTHOR CONTRIBUTIONS

**Study concept and design**: Xiang Quanyong, Wang Jiaqi, Dai He, Chen Chong, Zhang Yuqing, and Qin Yu.

**Acquisition of data**: Wang Jiaqi, Dai He, Ding Ganling, Xiang Quanyong, Zhang Yongqing, and Chen Chong.

**Analysis and interpretation of data**: Wang Jiaqi, Ding Ganling, Xiang Quanyong, Chen Chong, Qin Yu, and Zhang Yongqing.

**Drafting of the manuscript**: Ding Ganling, Chen Chong, Wang Jiaqi, Qin Yu, and Xiang Quanyong.

**Critical revision of the manuscript for important intellectual content**: Xiang Quanyong, Wang Jiaqi, Ding Ganling, Chen Chong, and Zhang Yuqing.

**Statistical analysis**: Wang Jiaqi, Dai He, Ding Ganling, Chen Chong, and Qin Yu.

**Obtained funding**: Xiang Quanyong.

**Technical or material support**: Zhang Yuqing, Zhang Yongqing, and Qin Yu.

**Study supervision**: Xiang Quanyong.

## Data Availability

The authors can provide the data on request.
